# A New Finite Element Simulation Methodology for Analyzing the Mechano-Electrochemical Effects of Al Alloys

**DOI:** 10.3390/ma19112307

**Published:** 2026-05-29

**Authors:** Hailiang Huang, Zhuzhu Zhang, Guixue Bian, Haitao Mao

**Affiliations:** 1 Qingdao Campus, Naval Aeronautics University, Qingdao 266041, China; 2 Unit 92728 of PLA, Shanghai 200040, China

**Keywords:** Al alloy, aircraft structural corrosion, corrosion simulation, reconstruction, mechano-electrochemistry

## Abstract

A new finite element simulation methodology for analyzing the mechano-electrochemical effects of Al alloys with intermittent measurement and reconstructed boundary conditions is proposed. It enables the simulation of the coupled mechano-electrochemical effects within the entire elastoplastic range of Al alloys. The model’s accuracy was verified through measurements of galvanic current, coupled potential, and corrosion morphology. This study indicates that the non-uniform stress distribution on a metal surface results in inconsistent electrochemical properties, leading to the spontaneous formation of anodes and cathodes and facilitating galvanic corrosion. Regions with stress concentration act as anodes in the corrosion reaction, while other areas serve as cathodes. The electrolyte domain is approximately polarized to the same potential, but there are also minor differences between different regions. As the stress concentration gradually increases, the mixed potential decreases, leading to greater polarization and an accelerated corrosion reaction rate. The galvanic current and the coupled potential calculated by the model differ from the measured values by less than 15%. Moreover, the observed corrosion morphology is consistent with the calculated results, indicating that the model provides good predictions of coupled mechano-electrochemistry.

## 1. Introduction

Aircraft always operate under extremely complex service environments, where humid atmospheric conditions, marine saline atmospheres and various industrial pol-lutants are widely prevalent. Environment-triggered corrosion is one of the primary causes of irreversible structural damage to aluminum alloy components in aviation, especially for aircraft long-term deployed at coastal airfields and performing continuous maritime flight missions [1,2,3,4,5,6]. Extensive studies have confirmed that the corrosion evolution of aircraft aluminum alloys highly correlates with external mechanical stress. Mechanical loading and environmental corrosion exhibit a synergistic coupling effects, which jointly accelerate the attenuation of the mechanical and anti-corrosion properties of materials, endangering the structural integrity and service safety of aircraft [6,7,8]. However, most existing relevant studies only independently discuss environmental corrosion’s characteristics or mechanical stress’s effects, with superficial qualitative descriptions of their coupling mechanisms. A comprehensive and in-depth critical analysis of the mechano-electrochemical coupling behavior covering full elastic–plastic deformation stages is still lacking, resulting in an insufficient understanding of the intrinsic mechanism through which the corrosion of aviation aluminum alloys accelerates under actual service conditions.

As early as the 1950s, Hoar [9] pioneered the exploration of coupled stress–corrosion behavior and verified that the plastic yield deformation of metallic materials can reduce the activation energy of anodic dissolution, thereby significantly boosting corrosion kinetics. On this basis, Hoar defined the mechano-electrochemical effect, establishing the basic understanding of stress-promoted corrosion and laying a preliminary theoretical foundation for subsequent research. Nevertheless, Hoar’s research had inherent limitations: it only qualitatively described stress-induced corrosion acceleration, without quantitative theoretical derivation, parametric analysis, or systematic verification of the different coupling laws under elastic and plastic deformation states, failing to reveal the essential mechanisms of deformation-dependent corrosion. In the 1990s, Gutman [10] proposed a theoretical mechano-electrochemical system based on dislocation evolution theory, which quantitatively explained the acceleration mechanism through which mechanical stress affects material corrosion dissolution, which has become the most widely adopted theoretical basis for current coupling research. Critically, this classical theory has prominent application defects. It relies heavily on a series of microscopic characteristic parameters that are extremely difficult to test and calibrate, including dislocation density and orientation correlation coefficients under plastic deformation, which make it basically inapplicable to plastic deformation under working conditions. Furthermore, the accuracy and universal applicability of Gutman’s theory in the pure elastic deformation interval have been controversial in academic circles. Multiple studies have pointed out that the theoretical prediction results deviate significantly from experimental data for aerospace aluminum alloy materials, and no unified and recognized improvement scheme has yet been formed [11,12].

Restricted by the limitations of in situ microscopic testing technologies, it is difficult to accurately capture the evolution of local corrosion’s morphology and the changes in regional electrochemical parameters in deformed materials. Consequently, numerical simulation has become the dominant method for investigating coupled mechano-electrochemical multi-field problems. Yan et al. [13] and Hussein Ali et al. [14] conducted targeted research on 7050 and 7075 aviation aluminum alloys, establishing finite element models of corrosion defects to clarify the differences in coupling rules under different deformation states. They concluded that mechanical-electrochemical coupling has a negligible impact on the evolution of corrosion in the elastic deformation stage, while local plastic deformation can sharply enhance regional electrochemical corrosion activity. This stress-dependent corrosion acceleration has been verified in ferrous alloys [15,16,17,18,19,20], stainless steels [21,22], and magnesium alloys [23,24,25], proving its universal material applicability. On the basis of single-point damage research, Wang [26] further optimized the numerical model, expanded the research object from isolated local damage to randomly distributed multi-point damage, and explored the corrosion failure mechanism of stiffened aircraft panels. However, all the above numerical models have a common critical defect: they adopt fixed corrosion boundary conditions and rigid interface assumptions, completely ignoring the dynamic morphological deformation of corrosion interfaces and the reverse feedback regulation effect on subsequent corrosion expansion. Such oversimplified modeling methods cannot reproduce the real-time iterative evolution process of corrosion and deformation in actual aircraft service environments, leading to inaccurate simulation results and limited engineering guiding value.

To compensate for the limitations of fixed interface modeling, scholars have attempted to introduce dynamic interface tracking algorithms to optimize coupling models. Funded by the U.S. Naval Research Office, Adlakha [25] and Bazehhour [27] constructed transient multi-field coupling models for dissimilar material joints, adopted the ALE dynamic interface tracking algorithm to characterize the galvanic corrosion propagation law under different loading modes, and dynamically predicted corrosion interface expansion. With the development of computational simulation technology, advanced implicit interface capture methods such as the phase-field method and the flow field dynamic analysis method have also been gradually applied to coupled mechano-electrochemical simulations [11,28,29,30,31]. Although these studies have realized the dynamic simulation of corrosion interfaces, they have failed to break through the core bottleneck of the existing theoretical systems. Almost all current coupled mechano-electrochemical simulation frameworks are bounded by classic Gutman theory and can only achieve the accurate simulation of material elastic deformation responses [10,15,32,33,34,35]. Once the material enters the plastic deformation stage, the unmeasurable microscopic parameters in the theory lead to invalid coupling calculations. Therefore, there is still a prominent research gap in coupled full-range multi-physics field simulation covering both the elastic and plastic deformation stages [17,18,32,33].

In this study, a custom-designed electrochemical testing system was independently developed for applications under stress loading conditions. A novel discontinuous method for the measurement and reconstruction of boundary conditions was constructed, which effectively circumvents the limitation of Gutman’s mechano-electrochemical theory: its poor applicability in the plastic deformation regime of materials. Subsequently, a new coupled mechano-electrochemical model was established. This model enables the numerical simulation of coupled mechano-electrochemical multi-physics fields for aluminum alloys over the entire elastic–plastic range, and the feasibility and reliability of the proposed method were experimentally validated.

## 2. Electrochemical Performance of 2A12 Al Alloy Under Stress

### 2.1. Preparation of Test Specimens and Design of Test Devices

**Figure 1:**
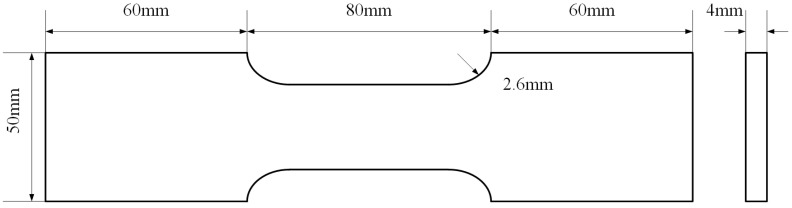
Schematic illustration of test specimen geometry and dimensions.

Using the 2A12-T4 Al alloy commonly employed in aircraft as the research subject, a “dog-bone”-shaped flat specimen was selected, with its specific parameters depicted in Figure 1. The chemical composition of 2A12 Al alloy is listed in Table 1.

**Figure 2:**
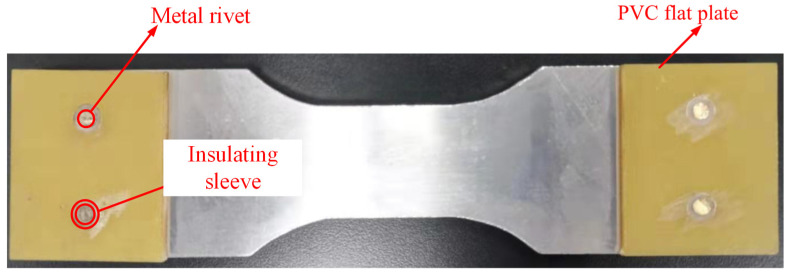
Specimens for evaluation of electrochemical performance under stress.

**Figure 3:**
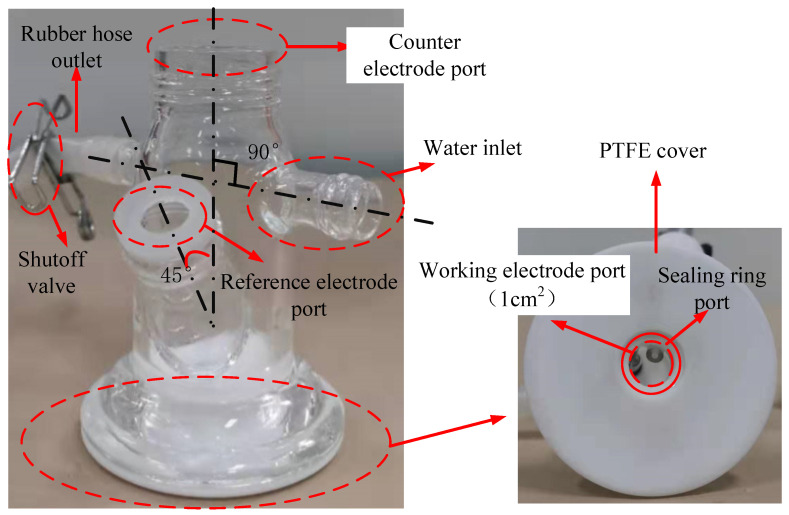
Custom-designed electrochemical cell for testing Al alloy under stress.

**Figure 4:**
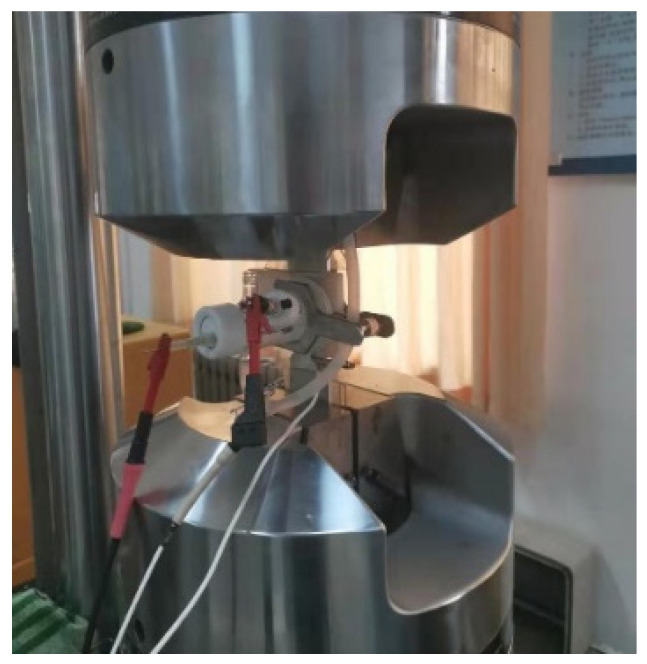
Electrochemical testing system under stress.

The tensile load was applied using an MTS810 fatigue testing machine (MTS Systems Corporation, Eden Prairie, MN, USA). Directly clamping the Al alloy specimen onto the MTS810 fatigue testing machine would introduce significant electrical interference during potentiodynamic polarization and electrochemical impedance spectroscopy measurements, thus compromising the accuracy of the obtained electrochemical data. To resolve this issue, it was essential to ensure electrical insulation between the Al alloy specimen and the testing machine fixture, while ensuring the specimen would not detach under stress loading conditions. For this purpose, a PVC plate was fixed to the Al alloy specimen using metal pins to form an integrated assembly, which ensured the structural stability of the specimen during stress loading. The insulating sleeve played a critical role in preventing electrical contact between the metal pins and the Al alloy specimen, which would otherwise have introduced additional electrical interference and have affected the reliability of the electrochemical tests (Figure 2). The electrochemical performance testing device for materials under stress utilized a custom-designed electrolytic cell (Figure 3) with a through-hole of 1 cm^2^ at the bottom. The cell was sealed with an O-ring and mounted laterally onto the specimen, as illustrated in Figure 4. Additionally, to avoid interference from external electromagnetic signals, the specimen was shielded from the surroundings using Al foil. Before conducting the electrochemical measurements, the specimen was sequentially polished with water-soaked sandpaper in the order of 240#, 800#, and 1500# grit to remove the surface oxide layer.

### 2.2. Measurement of Stress–Strain Curve and Selection of Electrochemical Measurement Points

**Figure 5:**
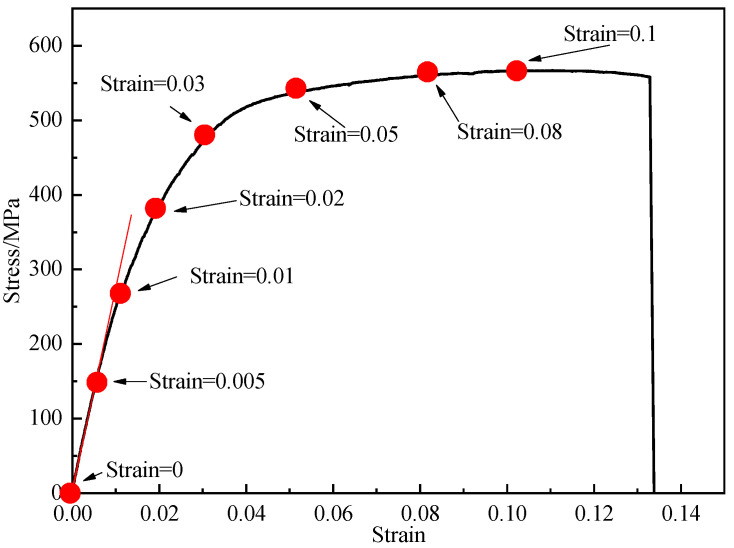
Stress–strain curve and selection of electrochemical measurement points.

Tensile tests were performed using an MTS810 fatigue testing machine at a 2 mm/min strain rate, while strain gauges were used to record the strain in the material. The resulting stress–strain curve is shown in Figure 5. The electrochemical measurements were conducted intermittently at eight specific strain points: 0, 0.005, 0.01, 0.02, 0.03, 0.05, 0.08, and 0.1. These points were selected to evaluate the electrochemical performance of Al alloy under different strain conditions, including measurements of EIS and polarization curves.

### 2.3. Electrochemical Testing and Results Under Different Strains

#### 2.3.1. Electrochemical Testing

A PARSTAT P4000 electrochemical workstation (Princeton Applied Research (AMETEK, Inc., Berwyn, PA, USA) was utilized to assess specimens’ polarization curves and EIS. The electrolyte solution was a 5 wt% NaCl (pH = 4). The testing was conducted using a three-electrode system, in which the specimen under test served as the working electrode (WE), a saturated calomel electrode (SCE) as the reference electrode (RE), and a Pt electrode as the counter electrode (CE). The open-circuit potential (OCP) was initially recorded during the measurement process. Once the OCP reached a stable state, indicated by a potential change of no more than 10 mV over a 300 s period, EIS measurements were conducted. The frequency range for EIS spanned from 10^5^ to 10^−2^ Hz, with seven sampling points per decade and a sinusoidal excitation amplitude of 10 mV. After the test, the EIS data were analyzed using ZSimpWin software 3.60. The potentiodynamic scanning method was employed for the polarization curve measurements, with a scanning range of −0.5~0.5 V (vs. OCP) and a scan rate of 1 mV/s. All polarization curves and EIS measurements were repeated three times to guarantee data repeatability and reliability.

#### 2.3.2. EIS Results

**Figure 6:**
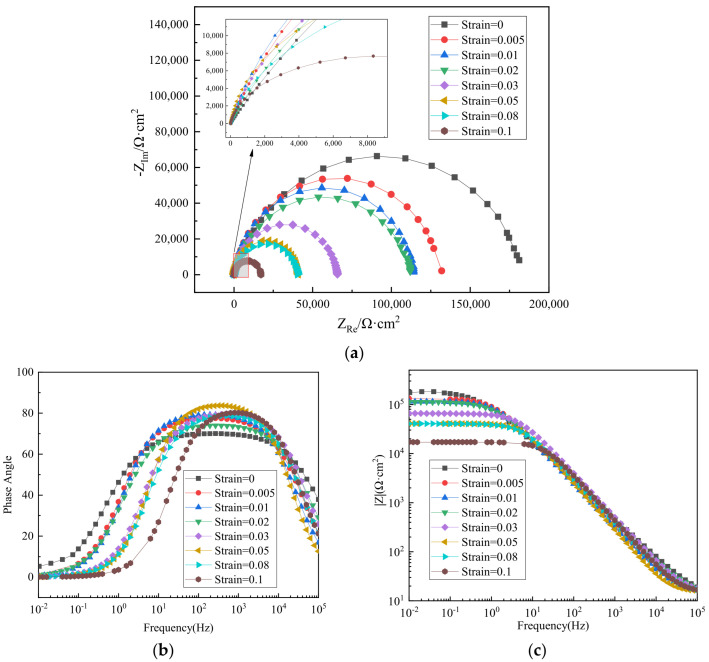
EIS of 2A12 Al alloy under different strain conditions. (**a**) Nyquist plot; (**b**) Phase angle plot; (**c**) Bode plot.

**Figure 7:**
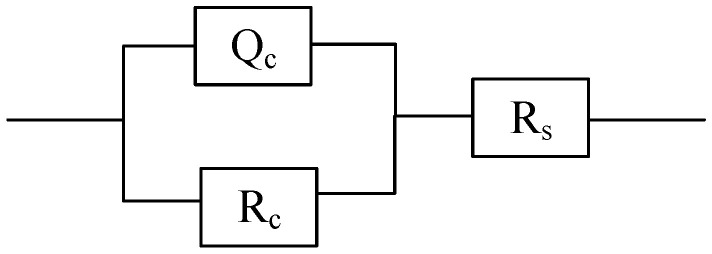
Equivalent circuit.

The EIS spectra of 2A12 Al alloy under different strain conditions are shown in Figure 6. As can be seen from the spectra, the EISs of the 2A12 Al alloy under different strain states are generally similar. All Nyquist plots exhibit a depressed semicircular characteristic with only one arc, and no significant diffusion impedance features are observed in the low-frequency region. In the Bode plot, $\mathrm{lg}\left|Z\right|-\mathrm{lg}f$ exhibits horizontal line characteristics in both the high-frequency and low-frequency regions. Between the high-frequency and low-frequency regions, $\mathrm{lg}\left|Z\right|-\mathrm{lg}f$ is a line with a negative slope. The peak phase angles under different strain conditions all exceed −π/4 and are less than −π/2, showing a single high-frequency peak. These results indicate that different tensile strains do not alter the corrosion mechanism of 2A12 Al alloy [36,37]. The EIS spectra exhibit only one time constant, and the current distribution is relatively uniform during testing. As a result, the test results are minimally affected by non-uniform current density distribution. Therefore, numerical fitting can be performed using the equivalent circuit shown in Figure 7 [15]. In this circuit, *R*_s_ represents the solution resistance. *Q*_c_ is a constant phase angle element that replaces the capacitive impedance behavior to eliminate the dispersion effect caused by non-uniform current distribution on the working electrode surface. *R*_c_ is the charge transfer resistance, representing the resistance to the electrochemical reactions at the anode and cathode, and is inversely proportional to the reaction rate [12].

The EIS parameters of 2A12 Al alloy under different strain conditions at open-circuit potential, obtained using Zsimpwin software fitting, are presented in Table 2. The fitted values of the dispersion coefficient *n* all fall within the range of (0.5, 1), indicating that the dispersion effects on the specimen surfaces vary under different strains. Therefore, it was appropriate to use a constant phase angle element instead of a pure capacitor in the equivalent circuit fitting [37]. The charge transfer resistance decreases progressively with increasing strain, indicating that the corrosion reaction becomes more likely to occur. This finding is consistent with Gutman’s mechano-electrochemical theory [10].

#### 2.3.3. Results of Polarization Curves

**Figure 8:**
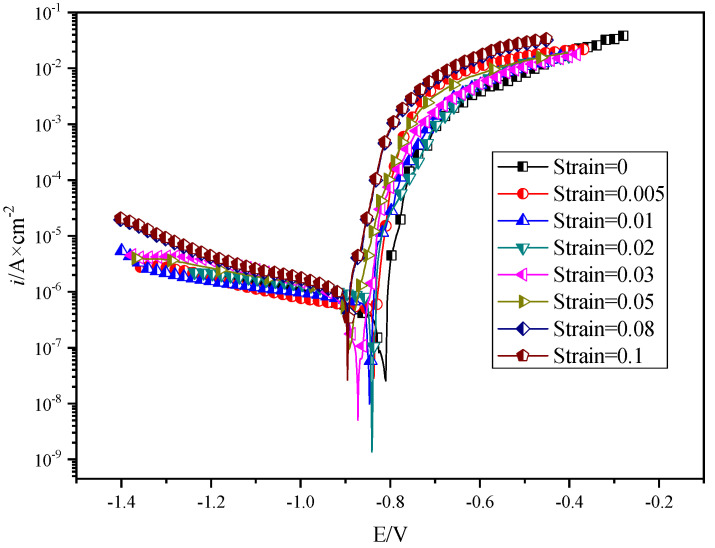
Polarization curves of 2A12 Al alloy under different strain conditions.

The polarization curves of 2A12 Al alloy under different strain conditions are shown in Figure 8. The similar shapes of the polarization curves indicate that the electrochemical reactions of the material remain unchanged under different strain conditions, which is consistent with the results of the EIS measurements [38]. After the polarization potential reaches the self-corrosion potential, the current density of the anodic reaction increases gradually with the potential, and no significant passivation phenomenon is observed. This suggests that the Al alloy remains in an active dissolution state [38,39].

The polarization curves were fitted using Cview software 2.6 to obtain the self-corrosion potential (*E*_corr_) and the self-corrosion current density (*i*_corr_), as shown in Table 3. The results indicate that as the strain increases, the self-corrosion potential (*E*_corr_) decreases, suggesting reduced corrosion resistance. When the material transitions from the elastic to the plastic regime, the self-corrosion potential drops sharply, and the corresponding self-corrosion current density (*i*_corr_) increases dramatically. However, the change in self-corrosion potential diminishes as plastic deformation progresses into the strain-hardening stage and even the initial stage of localized necking. In contrast, the self-corrosion current density continues to rise rapidly. This suggests that while loading reduces the material’s corrosion resistance, once the load reaches a certain level, the material’s susceptibility to corrosion does not change significantly, but the corrosion rate continues to increase. In contrast, the self-corrosion current density rapidly increases, indicating that the load reduces the material’s corrosion resistance. However, when the load increases to a certain extent, the material’s corrosion susceptibility does not change much, yet its corrosion rate continues to increase. According to Gutman’s mechano-electrochemical theory, the underlying reasons can be analyzed as follows: When metals undergo elastic deformation, tensile stress does not promote the activation or multiplication of dislocations. However, it can cause edge dislocations to emerge at the surface in areas with dislocation defects. This increases the surface energy of the metal, lowers the corrosion potential, and leads to preferential dissolution at the junctions between dislocations and the crystal surface [10,40]. Corrosion products preferentially nucleate in these regions, and, as tensile stress increases, the number of corrosion product nuclei gradually increases. When tensile stress exceeds the material’s elastic limit and plastic deformation occurs, it induces dislocations to slip on the metal’s surface. During this process, tensile stress promotes the multiplication and movement of dislocations. When dislocations encounter obstacles, dislocation pile-ups occur, leading to reorganization and annihilation. This releases stored energy in the metal, reflected in the open-circuit potential and self-corrosion current density [15,17].

## 3. Coupled Mechano-Electrochemical Simulation of Localized Damage

### 3.1. Mathematical Formulation of the Model

The isotropic strain hardening model is employed to characterize the elastoplastic behavior of Al alloy [13,17,18]:

(1) $$\sigma_{\mathrm{yhard}}=\sigma_{\exp}\left(\varepsilon_{\mathrm{eff}}\right)-\sigma_{\mathrm{ys}}=\sigma_{\exp}\left(\varepsilon_{p}+\frac{\sigma_{e}}{E}\right)-\sigma_{\mathrm{ys}}$$

where $\sigma_{\exp}$ is the experimentally measured stress–strain curve (Figure 5); $\varepsilon_{\mathrm{eff}}$ is the total effective strain; $\sigma_{\mathrm{ys}}$ is the yield strength, 380 MPa; $\varepsilon_{p}$ is the plastic strain; $\sigma_{e}$ is the elastic stress; *E* is Young’s modulus, 67.5 GPa; $\sigma_{e}/E$ is the elastic strain; Poisson’s ratio of 2A12 Al alloy is 0.33. The elastoplastic simulation follows the Von Mises yield criterion.

Assuming that the electrolyte solution is static, dilute and of uniform concentration, the convection and diffusion terms in the Nernst–Planck equation can be omitted. Further simplification of this equation yields the Laplace equation, which describes the potential distribution of the electrolyte (Equation (2)). The current density is subsequently calculated by means of Ohm’s law (Equation (3)) [5,41].

(2) $$\nabla^{2}\varphi_{l}=\Delta \varphi_{l}=0$$

(3) $$i_{l}=\sigma_{l}\nabla \varphi_{l}$$

where $\varphi_{l}$ is the electrolyte potential; $\sigma_{l}$ is the electrolyte conductivity, 5.6 S/m.

### 3.2. Three-Dimensional Reconstruction of the Polarization Curves

When subjected to stress, damaged areas within Al alloy can cause localized stress concentration, resulting in linear or non-linear stress distributions and strain on the material surface. According to Gutman’s mechano-electrochemical theory, the electrochemical performance of a material is closely related to its stress and strain. Therefore, the analysis of corrosion electrochemistry for load-bearing structures requires considering the coupled effects of stress and electrochemistry [11]. Since strain variations do not alter the mechanism of the material’s electrochemical reactions, this study introduces strain as an independent variable in the polarization curve (Equation (4)).

(4) i=f(η)⇒i=f(η,ε)

where *i* is the current density, *η* is the overpotential, and *ε* is the strain of the material.

**Figure 9:**
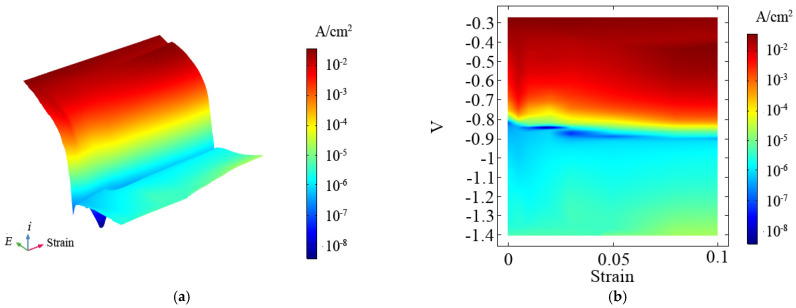
Reconstruction of the polarization curve. (**a**) Three-dimensional reconstruction of the polarization curves; (**b**) two-dimensional isosurface map of polarization curves.

By introducing the matrix concept and Lagrange interpolation, the conventional two-dimensional polarization curve is extended to a three-dimensional distribution (Figure 9). The interpolation is performed on the current density (not log-transformed) as a function of both potential and strain. The anodic and cathodic branches are treated separately to preserve their distinct electrochemical behaviors, and the overpotential is defined locally relative to the strain-dependent corrosion potential measured at each strain level. For interpolation calculation in this work, the built-in cubic Lagrange spline interpolation of COMSOL Multiphysics 6.2 was adopted, and linear extrapolation was used to process data outside the interval.

### 3.3. Mesh Generation and Boundary Conditions

**Figure 10:**
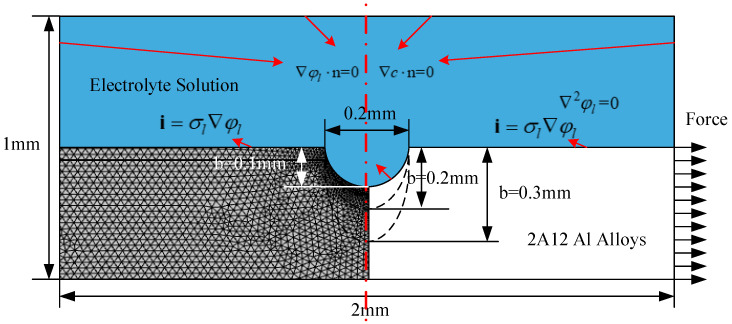
Model geometry, meshing, and boundary conditions.

Assuming that the local damage in Al alloy is an ideal hemisphere or ellipsoid, the model’s geometric configuration, mesh division, and boundary conditions are as shown in Figure 10. The boundary condition at the interface between the electrolyte and 2A12 Al alloy is defined by the reconstructed polarization curve. In contrast, the remaining boundaries of the electrolyte are assumed to be insulating. The model investigates the influence of tensile stress on the corrosion behavior of damage with the same surface area but different depths, specifically with depths (i.e., the ellipse’s central axis) of b = 0.1 mm, 0.2 mm, and 0.3 mm. To highlight the advantages of the intermittent measurement-based reconstruction of the polarization curve in corrosion simulations, a tensile force of F = 60 kN is applied to the side of the 2A12 Al alloy. This force exceeds the yield strength but remains below the tensile strength of the alloy, thereby ensuring a broad stress distribution across the metal cross-section without causing fracture.

### 3.4. Simulation Results of Localized Damage

#### 3.4.1. Coupled Distribution of Corrosion Field and Stress Field

(1) Coupled Stress–Potential Distribution

**Figure 11:**
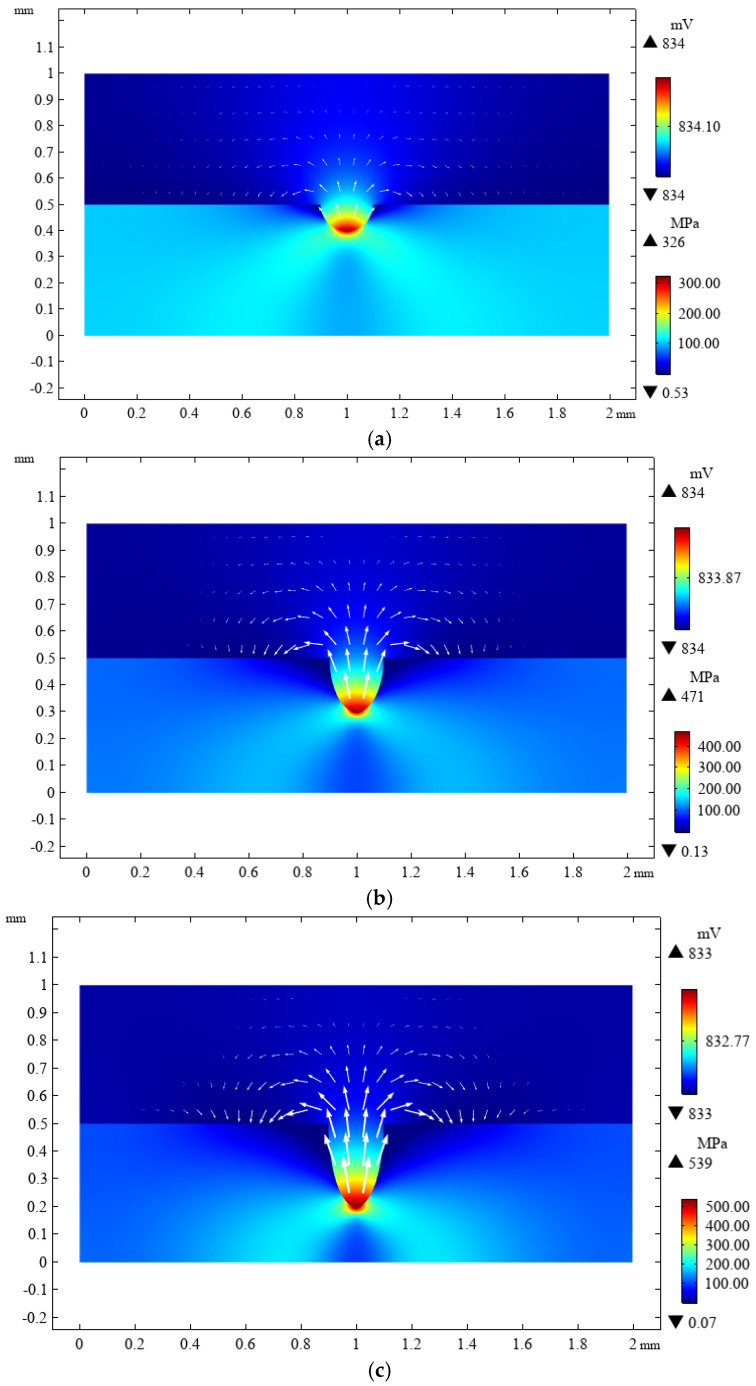
Coupled stress–potential distribution map. (**a**) b = 0.1 mm; (**b**) b = 0.2 mm; (**c**) b = 0.3 mm.

The stress–potential distributions for three damage depths (b = 0.1 mm, 0.2 mm, and 0.3 mm) are shown in Figure 11. Under stress, the damaged area exhibits significantly elevated stress compared with the surrounding regions. As the damage depth increases, the stress concentration becomes more pronounced, exceeding the material’s elastic limit and entering the plastic stage. However, it does not surpass the material’s fracture strength. Hence, no fracture occurs. The non-uniform stress distribution on the metal surface leads to inconsistent electrochemical performance, resulting in the spontaneous formation of anodic and cathodic regions and inducing galvanic corrosion. The white arrows in the figure, representing current flow in the electrolyte, indicate that the damaged area acts as the anode in the corrosion reaction. In contrast, the outer surface acts as the cathode. This is consistent with the literature [17]. The potential is approximately polarized to a similar level within the electrolyte domain, although minor differences exist between regions. As the damage depth increases, the mixed potential gradually decreases, and the degree of polarization increases, theoretically leading to a higher corrosion reaction rate. This explains why some metals that undergo uniform corrosion under stress-free conditions experience localized corrosion under stress, with more severe localized corrosion occurring as stress concentration increases [17,18].

(2) Coupled Strain–Current Density Distribution

**Figure 12:**
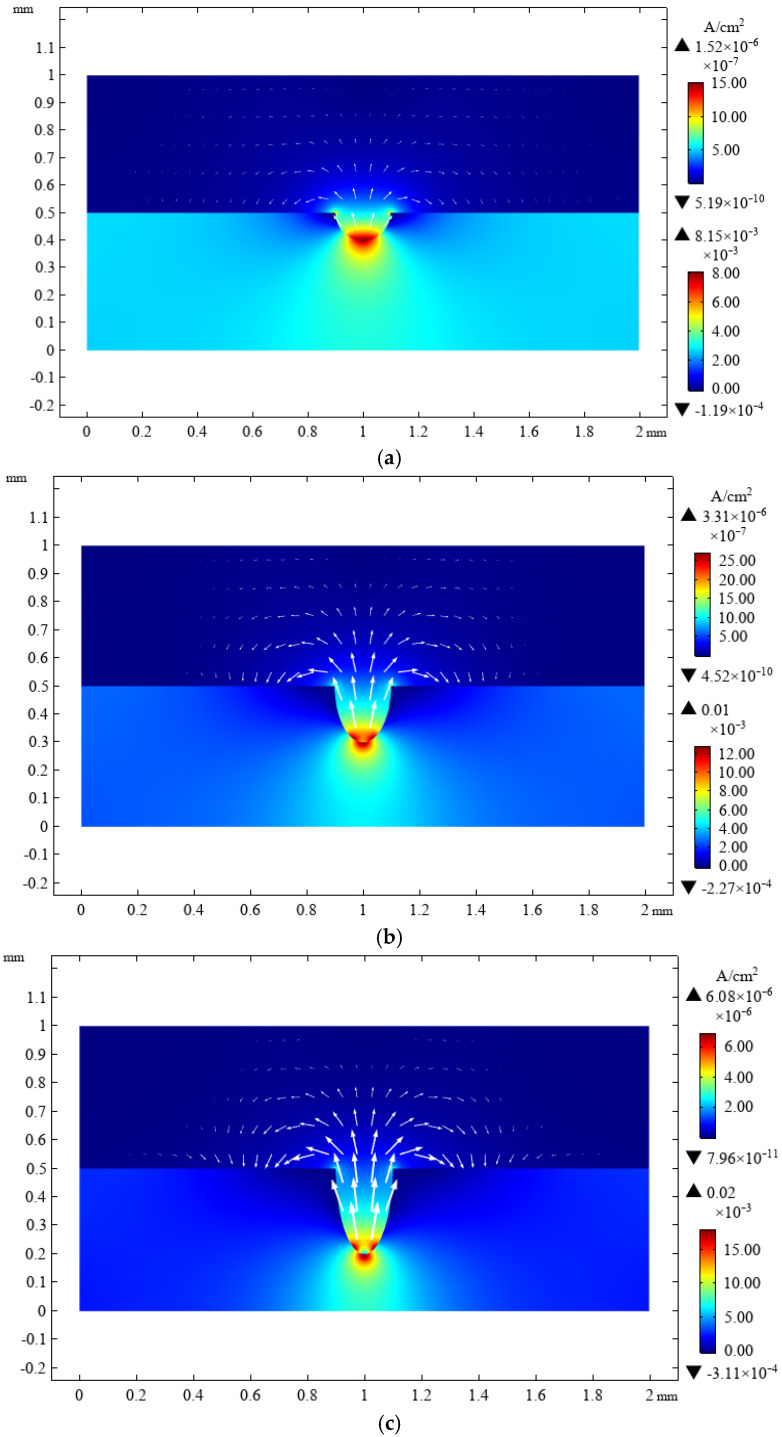
Coupled strain–current density distribution map. (**a**) b = 0.1 mm; (**b**) b = 0.2 mm; (**c**) b = 0.3 mm.

The strain–current density distributions obtained at three damage depths of b = 0.1 mm, 0.2 mm, and 0.3 mm are shown in Figure 12. It can be observed that the action of stress induces strain in the metal. The strain is most pronounced at the bottom of the damage for a given depth and increases as depth increases. The current density values within the electrolyte domain exhibit both positive and negative signs, indicating the spontaneous formation of anodic and cathodic regions at the metal–electrolyte interface. The maximum electrolyte current density occurs at the bottom of the damage. It is significantly higher than the material’s self-corrosion current density under the same strain. This indicates accelerated corrosion, which becomes more pronounced as the damage depth increases.

#### 3.4.2. Numerical Distribution of Coupled Corrosion and Stress Fields on Al Alloy Surfaces

**Figure 13:**
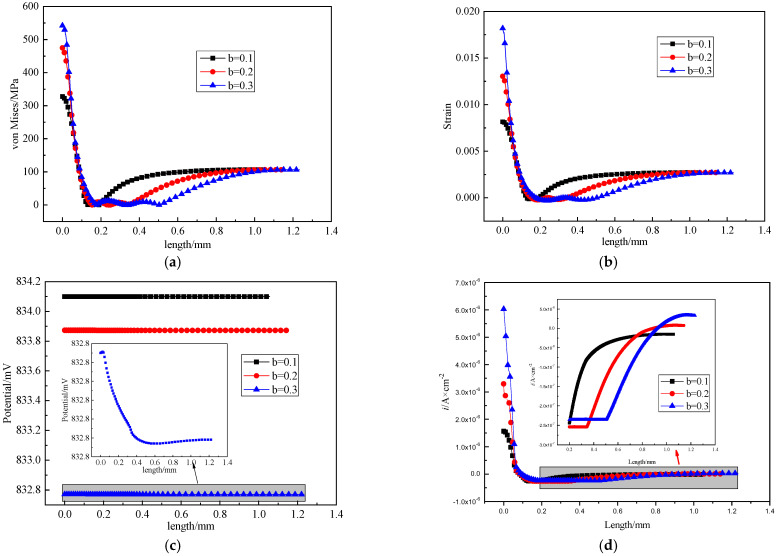
Numerical distribution map of Al alloy surface under coupled corrosion field and stress field. (**a**) Distribution of stress; (**b**) Distribution of strain; (**c**) Distribution of potential; (**d**) Distribution of current density.

The numerical distributions of the corrosion and stress fields at the electrode interface at three damage depths (b = 0.1 mm, 0.2 mm, and 0.3 mm) are shown in Figure 13. It should be noted that since the model is symmetric, the *x*-axis represents half the length of the electrode surface. At b = 0.2 mm and b = 0.3 mm, the bottom of the damage enters the plastic deformation stage, while the external regions of the damage remain in the elastic deformation stage for all three values of b.

Although the coupled potential within the electrolyte domain appears roughly uniform, magnifying it (Figure 13c) reveals distinct differences, with the highest potential at the bottom of the damage and relatively small variations on the external surface. The internal current density is predominantly positive, while the external current density is negative. This indicates that the internal region acts as the anode in the corrosion reaction, accelerating corrosion, while the external surface acts as the cathode, providing cathodic protection. The internal current density is several orders of magnitude higher than that under stress-free conditions and differs significantly from the current density values considering only stress, without accounting for the autocatalytic corrosion effect due to stress concentration [17,18]. This demonstrates that thew corrosion assessment of load-bearing structures in aircraft under stress-free conditions or local stress–corrosion coupling is insufficient. A comprehensive force analysis of the entire structure is necessary [1,4,13].

## 4. Coupled Mechano-Electrochemical Simulation of Al Alloy Plates with Pre-Notched Holes

### 4.1. Geometric Model and Boundary Conditions

**Figure 14:**
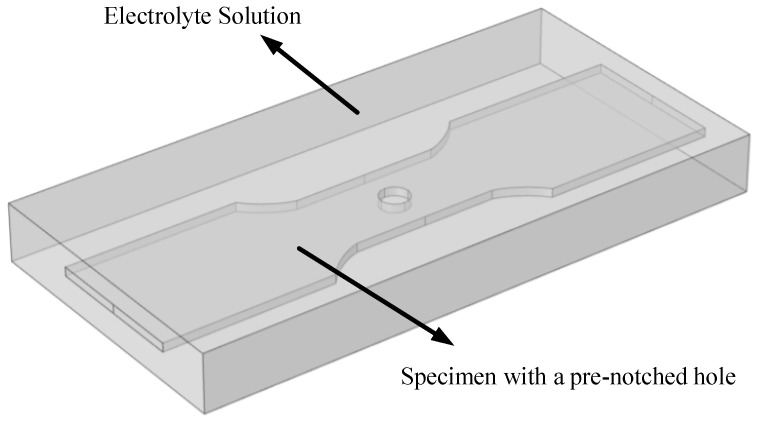
Geometric model of pre-notched plate specimen.

To simulate the effect of stress concentration at a hole on corrosion, a circular through-hole with a diameter of 1 cm was prefabricated in the middle of the specimen. The geometric model is shown in Figure 14. A uniform axial tensile force of 60 kN was applied to both sides of the specimen, ensuring a broad stress distribution range across the metal cross-section without causing fracture.

### 4.2. Simulation Results of Al Alloy Plates

**Figure 15:**
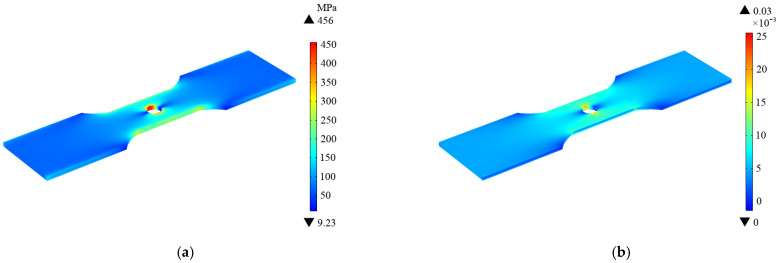

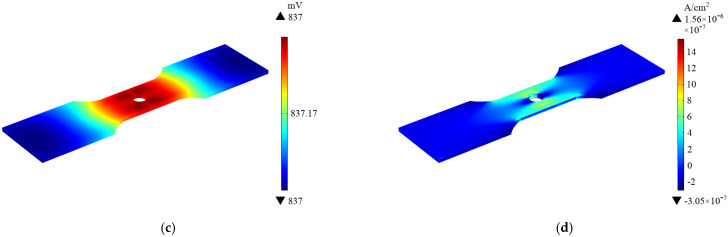
Stress-corrosion coupling map on an Al alloy plate with a pre-notched hole. (**a**) Distribution of stress; (**b**) Distribution of strain; (**c**) Distribution of potential; (**d**) Distribution of current density.

**Figure 16:**
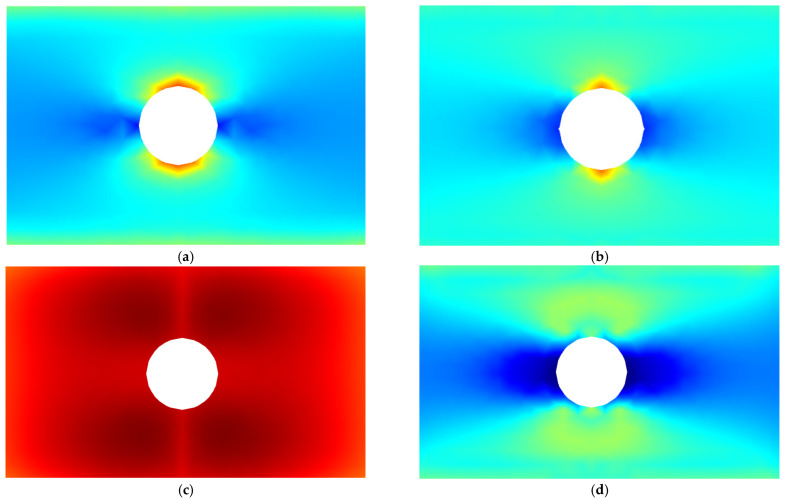
Detailed visualization of coupled stress–corrosion around hole. (**a**) Distribution of stress; (**b**) Distribution of strain; (**c**) Distribution of potential; (**d**) Distribution of current density.

The simulation results of the effect of load on the corrosion of a three-dimensional structure with a pre-notched hole are shown in Figure 15, with local details presented in Figure 16. Under the action of the load, the stress distribution on the specimen surface is extensive, with the area around the hole entering the plastic zone. However, the maximum stress does not exceed the material’s fracture strength; hence, no fracture occurs. The maximum strain on the specimen occurs at the edge of the hole perpendicular to the direction of stress (Figure 16b), which is consistent with the trends in the potential distribution (Figure 16c) and current density distribution (Figure 16d). This indicates that the presence of the load alters the corrosion propagation on the specimen surface. Comparing the strain distribution (Figure 15b) and current density distribution (Figure 15d) of the specimen with the self-corrosion current density values of the Al alloy under different strain conditions listed in Table 3, it is evident that the load intensifies the corrosion at the sites of stress concentration in the metal.

## 5. Experimental Verification

### 5.1. Corrosion Morphology

**Figure 17:**
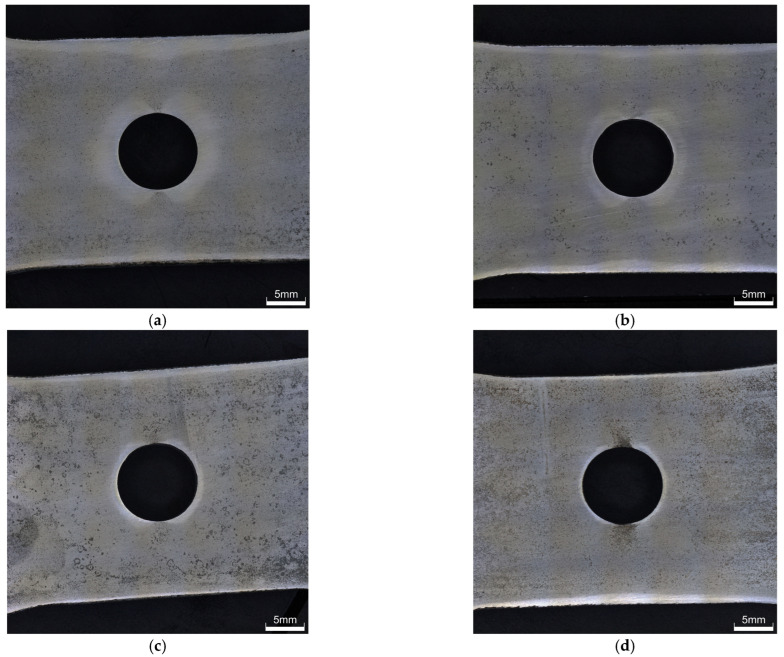
Corrosion morphologies under different cycles. (**a**) 10 d; (**b**) 20 d; (**c**) 30 d; (**d**) 40 d.

The Al alloy specimen with a pre-notched hole was subjected to a tensile stress of 60 kN, and its parallel section was immersed in a 5 wt% NaCl solution (pH = 4). The long-term validation experiment was carried out over four consecutive experimental periods, with each period lasting 10 days (totaling 40 days). The pH value of the solution was monitored with a calibrated pH meter every 2 d, and the solution was completely replaced with freshly prepared 5 wt% NaCl solution (pH = 4) once the measured pH deviated from the target value by more than ±0.2. After each cycle, corrosion products were removed according to GB/T 16545-2015 [42] by immersing the specimen in nitric acid at ambient temperature (20~25 °C) for 1~5 min. The corrosion morphology around the notched hole was then observed using an Olympus RH1000 microscope (Olympus Corporation, Tokyo, Japan), as shown in Figure 17.

The corrosion morphology around the notched hole is similar to the local details around the hole in Figure 16, with the most severe corrosion occurring in the region of the highest stress concentration and plastic deformation. This similarity confirms the model’s accuracy in predicting corrosion locations on the structure.

### 5.2. Galvanic Current and Coupled Potential

**Figure 18:**
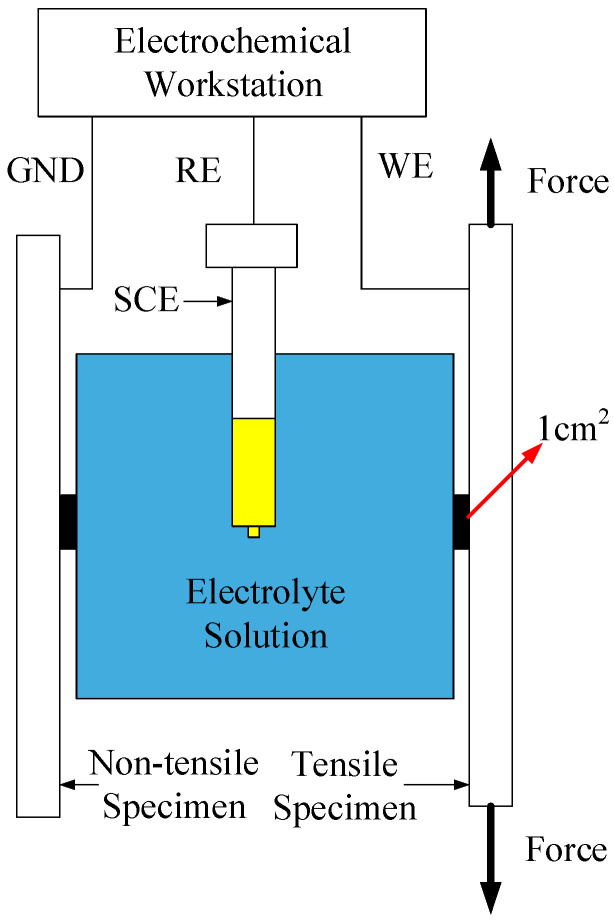
Schematic diagram of test verification device.

**Figure 19:**
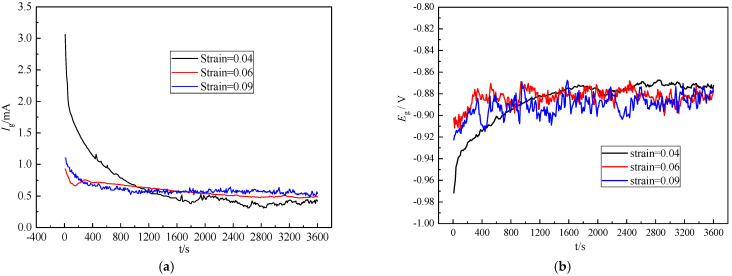
Measured values of galvanic current and coupled potential under various strains (0.04, 0.06, 0.09). (**a**) Galvanic current; (**b**) Coupling potential.

**Figure 20:**
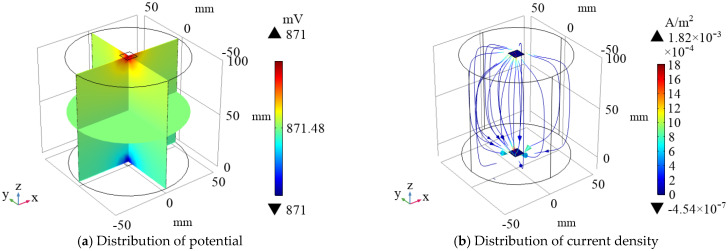
Simulation results of strain = 0.04 (**a**) coupled with strain = 0 (**b**).

Specimens with strain values of 0.04, 0.06, and 0.09 were subjected to static tensile testing and were paired with a blank specimen to form galvanic couples. These couples’ galvanic current and coupled potential were measured using an electrochemical workstation (PARSTAT P4000, Princeton Applied Research (AMETEK, Inc.), Berwyn, PA, USA), with the measurement setup shown in Figure 18. The measurement cycle was 3600 s, with a galvanic current value recorded every 10 s. The results of the galvanic current and coupled potential measurements are shown in Figure 19. Figure 20 shows the simulated coupled potential and galvanic current density distribution for a strain value of 0.04 and a blank specimen (i.e., a strain of 0). The galvanic current value was obtained by integrating the current density over the electrode surface shown in Figure 20b, and the same procedure was applied to the simulations for strains of 0.06 and 0.09. The average values of the galvanic current and coupled potential over the final 500 s of stabilized measurements were used as the reference values for each galvanic couple and compared with the simulated values, as shown in Table 4. The relative error *e* between the measured and simulated values of the galvanic current and potential was calculated using Equation (5) and is presented in Table 5.

(5) $$e=\left|\frac{n_{\mathrm{sim}}-n_{\mathrm{mea}}}{n_{\mathrm{mea}}}\right|\times 100\%$$

where *e* represents the relative error; *n*_sim_ and *n*_mea_ represent the simulated and measured values. The relative errors between the simulated and experimental values of the galvanic current and coupled potential are all within 15%. The error is relatively large only when the strain is 0.04, while it does not exceed 7% for the other cases. This may be due to the significant variation in the stress–strain curve within the strain range from 0.03 to 0.05, which causes considerable changes in the material’s electrochemical properties. Consequently, the polarization curve obtained through linear Lagrange interpolation may contain some errors. However, the errors generated by this method are still within an acceptable range.

## 6. Conclusions

(1) Different tensile strains do not alter the corrosion mechanism of 2A12 Al alloy. As the strain increases from 0 to 0.1, the charge transfer resistance (*R*_c_) decreases from 186.5 kΩ·cm^2^ to 17.15 kΩ·cm^2^, thereby facilitating corrosion reactions. The corrosion potential (*E*_corr_) shifts from −0.8118 V to −0.8953 V, whereas the corrosion current density (*i*_corr_) increases from 0.35554 μA·cm^−2^ to 1.15518 μA·cm^−2^, representing an approximate threefold increase. Upon the transition from the elastic to the plastic stage, *E*_corr_ decreases rapidly, and *i*_corr_ rises sharply; as plastic deformation intensifies and enters the strain-hardening stage, the variation in the amplitude of *E*_corr_ diminishes, while *i*_corr_ continues to increase. This indicates that the material’s tendency to corrode stabilizes once the strain exceeds a certain threshold, yet the corrosion rate continues to escalate.

(2) The non-uniform stress distribution induces inconsistent electrochemical properties across the material’s surface, spontaneously forming anodic zones at stress concentration sites and cathodic zones in low-stress regions, resulting in galvanic corrosion. For local damage structures, as the damage depth increases from 0.1 mm to 0.3 mm, the stress concentration at the damage bottom intensifies and enters the plastic deformation regime; the mixed potential gradually decreases; the current density at the damage bottom is significantly higher than that under either stress-free or uniform-strain conditions, exhibiting pronounced characteristics of local corrosion acceleration. For three-dimensional structures with prefabricated holes, the maximum strain occurs at the hole edge perpendicular to the loading direction; this location aligns with the distribution trends of both potential and current density and serves as the preferential region for corrosion propagation.

(3) A three-dimensional polarization curve reconstruction method based on intermittent measurement and Lagrange interpolation was proposed and implemented as the boundary condition for coupled multi-field simulation, thereby enabling the coupled multi-physics numerical simulation of mechano-electrochemical effects across the entire elastic–plastic range of the Al alloy. Experimental validation shows that the relative errors in the simulated galvanic current and coupled potential, compared with the measured values, are both within 15%. The corrosion morphology of specimens with prefabricated holes after 40 days of immersion under a 60 kN tensile load aligns with the simulated current–density distribution, confirming the model’s predictive capability for corrosion localization. In regions where the stress–strain curve exhibits pronounced variation (e.g., the strain interval of 0.03–0.05), increasing the density of measurement points is recommended to further enhance the interpolation accuracy and simulation reliability.

## Figures and Tables

**Table 1:** The chemical composition of 2A12 Al alloy.

Element	Cu	Mg	Mn	Si	Fe	Zn	Ti	Al
Content (wt%)	3.8~4.9	1.2~1.8	0.3~0.9	≤0.5	≤0.5	≤0.3	≤0.15	Bal.

**Table 2:** EIS fitting parameters for 2A12 Al alloy under various strain conditions.

Strain	0	0.005	0.01	0.02	0.03	0.05	0.08	0.1
*R_s_*/Ω·cm^2^	11.03	14.28	15.38	13.75	14.81	15.38	14.7	14.89
*R*_c_/kΩ·cm^2^	186.5	132.1	114.6	112.9	65.74	40.87	40.52	17.15
*Q*_c_/Ω^−1^·cm^−2^·s^−n^	2.19 × 10^−6^	1.36 × 10^−6^	1.29 × 10^−6^	1.54 × 10^−6^	7.56 × 10^−7^	8.36 × 10^−7^	9.93 × 10^−7^	6.77 × 10^−7^
*n*	0.7873	0.874	0.8956	0.8353	0.9014	0.9545	0.8978	0.929
χ^2^	4.55 × 10^−7^	5.56 × 10^−7^	1.37 × 10^−6^	2.06 × 10^−6^	8.75 × 10^−8^	5.12 × 10^−7^	1.48 × 10^−7^	6.37 × 10^−7^

**Table 3:** Self-corrosion potential (*E*_corr_) and self-corrosion current density (*i*_corr_) of 2A12 Al alloy under different strain conditions.

Strain	0	0.005	0.01	0.02	0.03	0.05	0.08	0.1
*E*_corr_/V	−0.8118	−0.8360	−0.8462	−0.8422	−0.8728	−0.8914	−0.8943	−0.8953
*i*_corr_/μA·cm^−2^	0.35554	0.45614	0.67626	0.86908	0.87364	0.9679	1.01375	1.15518

**Table 4:** Comparison of simulation and test results of galvanic current and coupling potential.

Strain	0.04	0.06	0.09
Simulation results *I*_g_/mA	0.4593	0.51743	0.53066
Test results *I*_g_/mA	0.40388	0.48397	0.54085
Simulation results *E*_g_/mV	−871.48	−887.97	−891.44
Test results *E*_g_/mV	−883.152	−886.126	−887.7

**Table 5:** Relative error (*e*) of simulation and test of galvanic current and coupling potential.

	Strain	0.04	0.06	0.09
*e*	Galvanic current *I*_g_	0.1372	0.0691	0.0188
	Coupled potential *E*_g_	0.0132	0.0021	0.0042

## Data Availability

The original contributions presented in this study are included in the article. Further inquiries can be directed to the corresponding authors.
